# Synergy between Ursolic and Oleanolic Acids from *Vitellaria paradoxa* Leaf Extract and β-Lactams against Methicillin-Resistant *Staphylococcus aureus:* In Vitro and In Vivo Activity and Underlying Mechanisms

**DOI:** 10.3390/molecules22122245

**Published:** 2017-12-16

**Authors:** Lucy Catteau, Nathalie T. Reichmann, Joshua Olson, Mariana G. Pinho, Victor Nizet, Françoise Van Bambeke, Joëlle Quetin-Leclercq

**Affiliations:** 1 Pharmacognosy Research Group, Louvain Drug Research Institute, Université catholique de Louvain, 1200 Brussels, Belgium; joelle.leclercq@uclouvain.be; 2 Cellular and Molecular Pharmacology Research Group, Louvain Drug Research Institute, Université catholique de Louvain, 1200 Brussels, Belgium; francoise.vanbambeke@uclouvain.be; 3 Bacterial Cell Biology Laboratory, Instituto de Tecnologia Química e Biológica António Xavier, Universidade Nova de Lisboa, 2780-157 Oeiras, Portugal; nreichmann@itqb.unl.pt (N.T.R.); mgpinho@itqb.unl.pt (M.G.P.); 4 Department of Pediatrics, University of California, San Diego, La Jolla, CA 92093-0760, USA; jolson@ucsd.edu (J.O.); vnizet@ucsd.edu (V.N.); 5 Skaggs School of Pharmacy and Pharmaceutical Sciences, University of California, San Diego, La Jolla, CA 92093-0760, USA; 6 MASSMET Platform, Louvain Drug Research Institute, Université catholique de Louvain, 1200 Brussels, Belgium

**Keywords:** *Vitellaria paradoxa*, ursolic acid, oleanolic acid, triterpenic acid, MRSA, β-lactamases, PBP2, skin infection

## Abstract

Combining antibiotics with resistance reversing agents is a key strategy to overcome bacterial resistance. Upon screening antimicrobial activities of plants used in traditional medicine, we found that a leaf dichloromethane extract from the shea butter tree (*Vitellaria paradoxa*) had antimicrobial activity against methicillin-resistant *Staphylococcus aureus* (MRSA) with further evidence of synergy when combined with β-lactams. Using HPLC-MS, we identified ursolic (UA) and oleanolic acids (OA) in leaves and twigs of this species, and quantified them by HPLC-UV as the major constituents in leaf extracts (21% and 6% respectively). Both pure triterpenic acids showed antimicrobial activity against reference and clinical strains of MRSA, with MICs ranging from 8–16 mg/L for UA to 32–128 mg/L for OA. They were highly synergistic with β-lactams (ampicillin and oxacillin) at subMIC concentrations. Reversion of MRSA phenotype was attributed to their capacity to delocalize PBP2 from the septal division site, as observed by fluorescence microscopy, and to disturb thereby peptidoglycan synthesis. Moreover, both compounds also inhibited β-lactamases activity of living bacteria (as assessed by inhibition of nitrocefin hydrolysis), but not in bacterial lysates, suggesting an indirect mechanism for this inhibition. In a murine model of subcutaneous MRSA infection, local administration of UA was synergistic with nafcillin to reduce lesion size and inflammatory cytokine (IL-1β) production. Thus, these data highlight the potential interest of triterpenic acids as resistance reversing agents in combination with β-lactams against MRSA.

## 1. Introduction

*Vitellaria paradoxa* C.F. Gaertn. (Sapotaceae), also called “shea butter tree”, is a tree that grows up to 14 m high. This tree is generally protected because of the economic value of shea butter, the fat extracted from the fermented kernel [1]. The leaves, roots, fruits, and stem bark of *Vitellaria paradoxa* (VP) have been used in traditional medicine to treat various infections [2,3,4]. This time-tested clinical application motivated us to investigate the leaves of this plant for antibacterial compounds and resistance modifying agents, focusing on skin infections caused by *Staphylococcus aureus*.

*S. aureus* is one of the most prominent human pathogens and a severe threat to public health with β-lactam antibiotics traditionally considered as a first line of treatment [5]. However, over time, *S. aureus* developed two main resistance mechanisms against this class of antibiotics. First, the production of β-lactamases inactivating these drugs, which was circumvented by the development of biopharmaceutical compounds resistant to enzymatic hydrolysis such as oxacillin or nafcillin. Second, exemplified by methicillin-resistant *S. aureus* (MRSA), the production of PBP2A, a cell wall transpeptidase and penicillin-binding protein (PBP), encoded by the *mecA* gene, with a decreased affinity for methicillin and most other β-lactam drugs [6]. This mechanism confers resistance to all β-lactam antibiotics, with the noticeable exception of cephalosporins from the fifth generation like ceftaroline or ceftobiprole recently brought to the market [7]. There thus exists an urgent need to find alternative and original strategies to fight against β-lactam-resistant staphylococci. In this context, it is of interest to restore the utility of antibiotics made less potent by the development of resistance by combining them with resistance modifying agents. One potential source of such compounds could be plant extracts [8].

In this work, we studied the direct and indirect (in combination with β-lactams) antimicrobial activity of the main components of *Vitellaria paradoxa* (VP) leaf extract against *S. aureus* and analyzed their potential mechanism of action.

To date, the antimicrobial activity of VP stem bark and leaf extracts has only been reported on strains of *S. aureus* using an agar diffusion method and high extract concentrations (50 g/L) [2,9]. However, during a preliminary screening of the direct and indirect antimicrobial activity of plants used in traditional medicine in Benin, we found that a VP leaf dichloromethane extract showed the best direct and indirect antimicrobial activities with a MIC = 250 mg/L and fractional inhibitory concentration indices (FICI) = 0.14–1 and 0.51–1 in combination with ampicillin and oxacillin, respectively, against a strain of MRSA [10]. In the present study, we continued our work on the dichloromethane extracts and identified, for the first time in leaves and twigs, oleanolic (OA) and ursolic (UA) acids as bioactive compounds. We quantified these two substances in extracts and studied the direct and indirect antimicrobial activities of these pure compounds in combination with β-lactams (ampicillin, oxacillin or nafcillin) on a panel of *S. aureus* reference strains as well as clinical MRSA isolates. We further explored the potential mechanism of action underlying this synergistic combination focusing on the potential effect of those compounds on the two principal β-lactam resistance mechanism of MRSA: β-Lactamases and PBP2A. Finally, we assessed the effectiveness of the combination of UA and nafcillin using an in vivo murine skin infection model.

## 2. Results and Discussion

### 2.1. Analysis and Quantification of Major Components of the Vitellaria paradoxa Crude Extract

**Figure 1:**
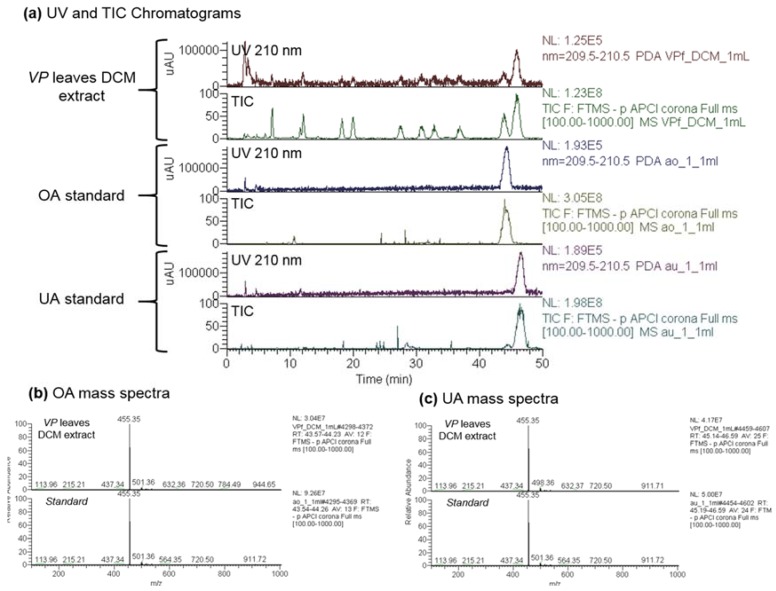
UV (210 nm) and Total Ion Current (TIC) Chromatograms of the *Vitellaria paradoxa* (VP) leaves dichloromethane (DCM) extract and of ursolic (UA) and oleanolic (OA) acids standards (**a**). Mass spectra of standards and of the peaks at the same retention times in the chromatogram of VP leaves DCM extract than OA (**b**) and UA (**c**).

Building on previous data [10], we began by identifying the active components of *Vitellaria paradoxa* crude extracts. LC/MS analysis of the crude dichloromethane leaf extract allowed identifying its two major components, UA and OA, by comparison of retention times, mass spectra and fragmentations with standard compounds. Chromatograms of this dichloromethane extract and of OA and UA standards, using the quantification MS compatible method with a 210 nm UV detection (Figure 1a UV) or the mass spectrometry detection (Figure 1a TIC), are shown in Figure 1. The correspondence between the mass spectra of standards and the corresponding peaks in the extract are shown in Figure 1b,c. Both triterpenic acids, which are widely distributed in the plant kingdom, are also present in the dichloromethane twig extract but not in the hexane extracts. Quantitative analysis revealed that the dichloromethane leaf extract contained 21.18 ± 0.50% of UA and 6.21 ± 0.12% of OA, vs. 2.06 ± 0.18% and 0.18 ± 0.04% respectively, in the twigs. While many other triterpenic acids have been identified in low amounts in *Vitellaria paradoxa* [11,12], this work is the first to report ursolic and oleanolic acids as the most abundant triterpenic acids in the leaves of *Vitellaria paradoxa.*

### 2.2. Antimicrobial Activity of the Crude Extract’s Major Components

Having identified the main components of VP crude extracts, we assessed the direct and indirect antimicrobial activity on MRSA (reference strain ATCC33591 and four clinical isolates) of the pure compounds to determine if they could account for the antimicrobial activity of the leaf extract (MIC 250 mg/L [10]). As shown in Table 1, both triterpenic acids were active against MRSA strains with MICs ranging from 8–16 mg/L for UA and 32–128 mg/L for OA, respectively. These values, at least for UA, are lower than the MIC of ampicillin or oxacillin against these strains.

Looking for resistance reversing agents, we combined each of the two triterpenic acids with two different β-lactams, namely ampicillin and oxacillin, in order to evaluate their indirect antimicrobial activity. These two antibiotics were selected because they differ in their susceptibility to MRSA resistance mechanisms. While both ampicillin and oxacillin are affected by PBP2A-mediated resistance, oxacillin resists to the action of β-lactamases, thanks to the steric hindrance brought by its voluminous lateral chain [13,14]. A decrease in the MIC of ampicillin could therefore result from an inhibition of either PBP2A mediated-resistance or of β-lactamase activity, while a decrease in oxacillin MIC would reflect an inhibition of the PBP2A-mediated resistance. As expected, all MRSA strains were resistant to both antibiotics (MICs higher than 32 mg/L), according to the European Committee on Antimicrobial Susceptibility testing (EUCAST) interpretative criteria [15]. UA and OA were synergistic with both ampicillin and oxacillin (FICI ranges including values ≤0.5), suggesting that at least PBP2A-mediated resistance was reverted in the presence of triterpenic acids (Table 1; see also isobolograms constructed from these checkerboard experiments and showing the synergistic interactions between ampicillin or oxacillin and both triterpenic acids in Figure S1).

**Figure 2:**
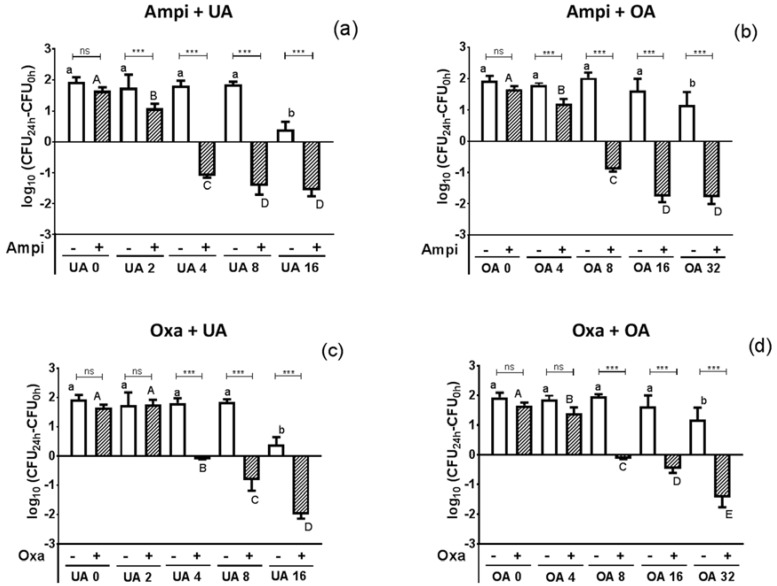
Change in bacterial inoculum after 24 h of culture of MRSA ATCC33591 in the presence of ampicillin (top panels **a**,**b**) or oxacillin (bottom panels **c**,**d**) at their MIC and used alone (open bars) or combined with UA (left panels **a**,**c**) or OA (right panels **b**,**d**) at the indicated concentrations (mg/L). Data are expressed as changed (in log_10_ CFU/mL) from the initial inoculum. Values are means ± SD of three independent experiments performed in duplicate. Statistical analyses: comparison between different concentrations of triterpenic acid (one-way ANOVA with Tukey post-hoc test): Data with different letters are significantly different from one another (*p* < 0.05; small letters: triterpenic acid alone; capital letters: Combination with the β-lactam). Comparison between triterpenic acid alone at a given concentration and in combination (*t*-test): *** *p* < 0.001; ** *p* < 0.01; * *p* < 0.05; ns, *p* > 0.05.

To further assess the potential of UA and OA to increase the activity of ampicillin or oxacillin, we performed time-kill assays on reference strain MRSA ATCC33591. The change in the log_10_ CFU/mL from the initial inoculum over time is shown in the supplementary material (Figures S2 and S3), with data obtained after 24 h of incubation reported in Figure 2.

Antibiotics alone at their MIC did not affect the growth of bacteria after 24 h; UA and OA alone reduced bacterial growth only at the highest concentration tested (16 and 32 mg/L, respectively). In combination, both triterpenic acids caused a concentration-dependent increase in antibiotic activity, which became significant for concentrations of 2 and 4 mg/L of UA and OA respectively in combination with ampicillin (panels a,b) and of 4 and 8 mg/L, respectively, when combined with oxacillin (panels c,d). A synergistic effect (≥2 log_10_-fold reduction in inoculum as compared to the antibiotic alone) was obtained for concentrations of 4 and 8 mg/L of UA and OA (i.e., ¼ MIC), respectively with both antibiotics. At the highest triterpenic acid concentrations tested, a 1.5–2 log_10_ decrease from initial inoculum was observed for both β-lactams, indicating that the presence of large concentrations of UA and OA in the VP leaf extract could explain the previously described direct and indirect antimicrobial activity against MRSA exposed to this extract alone or combined with β-lactams [10]. Conversely, the absence of activity for the twig extract could be attributed to its reduced content in these compounds. Previous studies from other groups have also documented antibiotic effects for these triterpenic acids against many bacterial species, their activity being higher against Gram-positive than Gram-negative bacteria and UA being more potent than OA (see [16,17] for recent reviews), as also observed here, yet divergent results have been reported regarding their activity on resistant bacteria, with Fontanay et al. describing no activity on MRSA or vancomycin-resistant enterocci (VRE) and Horiuchi et al. showing that both compounds remained active on both types of strains [18,19]. Their synergism with β-lactams has also been previously established, but only for UA combined with ampicillin against MRSA [20] and for both UA and OA combined with oxacillin against a fully susceptible MSSA [21]. We systemize here these effects, showing that synergy is observed for both compounds combined with either ampicillin or oxacillin against MRSA (including clinical isolates), which are of higher interest for clinical applications than MSSA.

### 2.3. Effect of OA and UA on PBP2 Recruitment to the Septum

**Figure 3:**
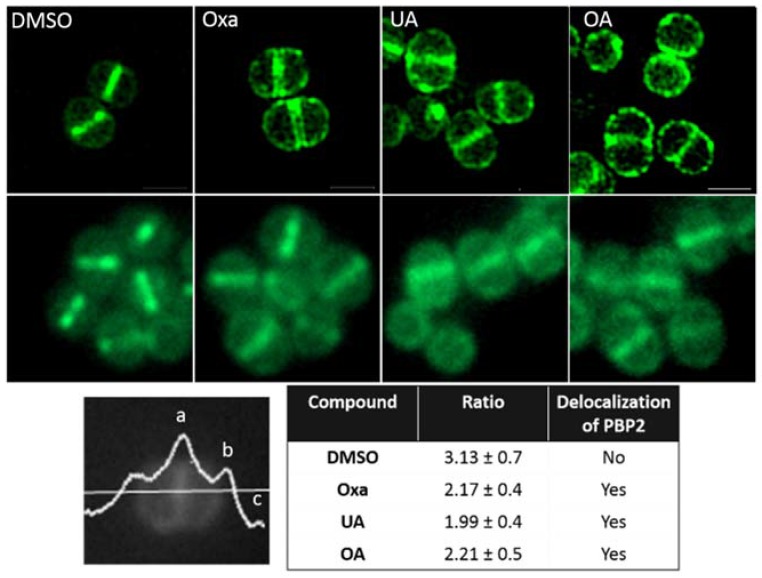
Influence of oxacillin and triterpenic acids on PBP2 localization. MRSA BCBPM073 carrying a gfp-pbp2 fusion was grown in TSB and incubated during 30 min without (DMSO) or with 1× MIC of Oxacillin (Oxa), UA or OA and observed by conventional (lower panel) and superresolution structured illumination (upper panel) microscopy. The localization of PBP2 was determined by calculating the fluorescence ratio (FR) (**a**–**c**)/(**b**,**c**) where “**a**” corresponds to the fluorescence found at the septum, “**b**” to the fluorescence of the peripheral membrane and “**c**” to the background fluorescence [23], as illustrated in the lower part of the figure. Values are the means ± SD from measurements of fluorescence ratios of 200 cells in each sample. A FR value >2 indicates an enrichment at the septum [where membranes from two adjacent daughter bacteria are apposed] and values ≤2, a localization spread over the entire membrane. Statistical analysis: one-way ANOVA with Dunnett’s post hoc test: FR values for bacteria exposed to Oxa, UA, or OA were different (*p* < 0.01) from the control.

The fact that OA and UA synergize with both ampicillin and oxacillin indicates that they may revert resistance mediated by PBP2A. This additional PBP is thought to maintain transpeptidase activity in the presence of β-lactams, and works in concert with the transglycosidase PBP2, which continues to transglycosylate the peptidoglycan. As no MRSA carrying a functional GFP-PBP2A has been engineered, we instead assessed the localization of PBP2 using a GFP-PBP2 fusion in MRSA strain COL by both conventional and structured illumination microscopy (Figure 3). Similar to previously published data, PBP2 preferentially localized to the septum (FR _(septum/peripheral membrane)_ = 3.13), while the addition of oxacillin, used here as a positive control, caused its redistribution over the entire cell membrane, which impairs the synthesis of new peptidoglycan at the cell division site [22]. The addition of both UA and OA also caused a delocalization of PBP2, suggesting that these compounds could also affect cell wall synthesis. Western blot analyses of total protein extracts of similarly treated COL GFP-PBP2 cells did not show consistent modifications in PBP2 or PBP2A expression, when compared to the control (data not shown).

### 2.4. Evaluation of β-Lactamase Activity

**Figure 4:**
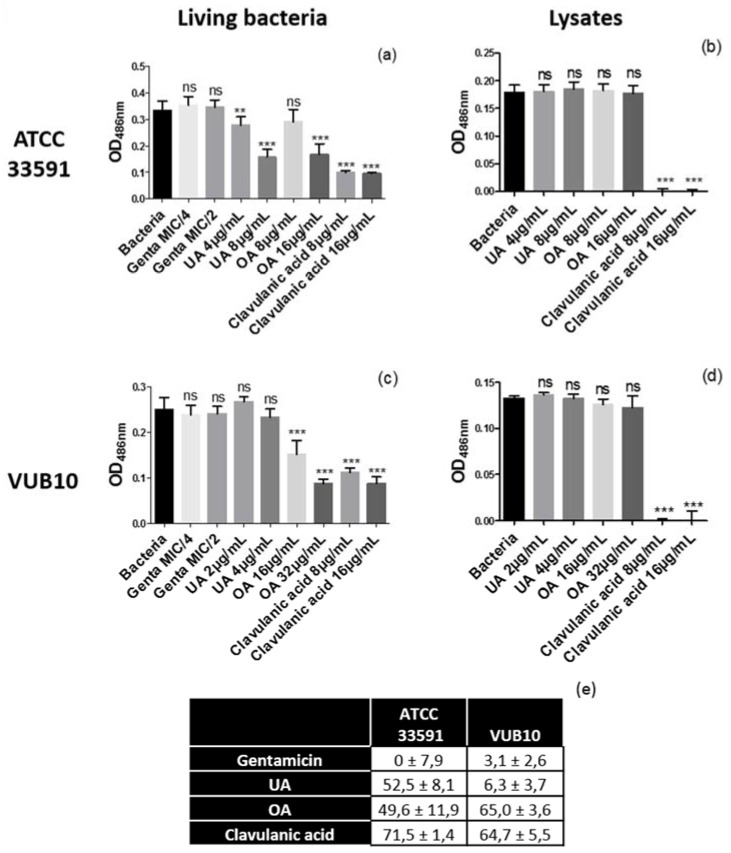
β-lactamase activity in intact (left panels) or lysed (right panels) MRSA ATCC33591 (upper panels) or VUB10 (lower panels) incubated during 30 min in control conditions or in the presence of gentamicin (negative control), clavulanic acid (positive control), UA, or OA at concentrations corresponding to their MIC/2 and MIC/4. Values are means ± SD of three independent experiments performed in duplicate. Statistical analyses were performed (two-way ANOVA with Dunnett’s post-test comparing each condition to the control «Bacteria» [*** *p* < 0.001; ** *p* < 0.01; * *p* < 0.05; ns, *p* > 0.5]). The Table below the graphs shows the percentages of inhibition of β-lactamase activity in living bacteria for ATCC33591 and VUB10 for agents used at concentrations corresponding to MIC/2 (*n* = 6).

The experiments performed in Figure 1 and Figure 2 did not allow excluding a concomitant reversion of β-lactamase-mediated resistance by triterpenic acids. We therefore examined the capacity of UA and OA to inhibit β-lactamases by following their influence on the rate of hydrolysis of the β-lactamase chromogenic substrate nitrocefin. These experiments were performed using intact bacteria and bacterial lysates of both the reference strain ATCC33591 and the clinical isolate VUB 10 (Figure 4).

As expected, gentamicin (protein synthesis inhibitor; MIC = 0.125 mg/L) did not inhibit β-lactamase activity in live bacteria neither in bacterial lysate, while clavulanic acid, a known inhibitor of β-lactamase activity, caused a marked decrease in nitrocefin hydrolysis in live bacteria and fully inhibited its degradation in bacterial lysates at subMIC concentrations. In contrast UA and OA at the highest concentration tested reduced nitrocefin hydrolysis in whole cells (panel a), but not in bacterial lysates (panel b) from ATCC33591. Similar results were obtained for OA with the clinical isolate VUB10 but not for UA (panels c,d), possibly due to the lower concentration tested (higher concentrations would have affected bacterial viability). Thus, these results suggest that these triterpenic acids, as opposed to clavulanic acid, indirectly impair β-lactamase activity, as their effect requires the machinery of a living cell.

Until now, few studies have investigated the mechanism by which UA and OA exert their antibacterial effects, often suggesting that they target the bacterial membrane or the peptidoglycan [21,23]. Due to their lipophilic character, UA and OA can accumulate in biological membranes, resulting in swelling and increased fluidity, as demonstrated using liposomes reconstituted from bacterial lipids [24]. In the present work, we show that both triterpenic acids revert β-lactam resistance mediated by PBP2A expression and by β-lactamases. Although we did not fully establish the underlying mechanisms, we provide some evidence that it probably requires an interaction of the triterpenic acids with the bacterial membrane. First, we show that both UA and OA are capable of delocalizing PBP2 in the membrane. This mechanism is critical for reversing the MRSA phenotype, which depends on the cooperation between PBP2 and PBP2A to carry out peptidoglycan biosynthesis and cross-linking [25]. It has been demonstrated for other adjuvants like the FtsZ inhibitor PC190723 [26], the oxacillin potentiators DNAC-1 and DNAC-2 [27,28] as well as for other natural compounds possessing a lipophilic moiety that allows them to intercalate in the bacterial membrane, like (−)-epicatechin gallate, a flavonoid from green tea [29,30]. Second, we also show that UA and OA interfere with β-lactamase activity, but do not act as competitive inhibitors of the soluble enzyme (as previously published [31]), neither as suicide substrates like clavulanic acid. In this respect, their effect contrasts with that of (−)-epicatechin gallate, which has been described as a direct inhibitor of penicillinase from *S. aureus* [32]. The fact that β-lactamase inhibition is observed only towards living bacteria suggests therefore an indirect effect of UA and OA, possibly related to their insertion in the bacterial membrane, the mechanism of which remains to be established.

### 2.5. Murine Model of Subcutaneous Infection

Despite uncertainties regarding the molecular mode of action of UA and OA, we investigated whether the synergistic effect of triterpenic acids with β-lactams was also observed in vivo. To this effect, we tested a combination of a triterpenic acid and nafcillin in a murine model of subcutaneous MRSA infection by the strain MRSA USA300 UAMS1182.

**Figure 5:**
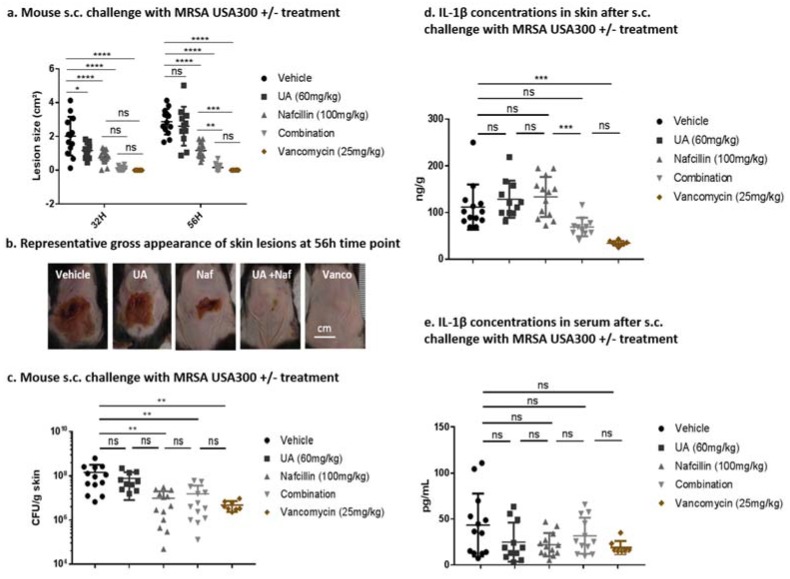
Antibacterial effects of UA in combination with nafcillin in a murine model of skin infection. Female c57/bl6 mice were infected with MRSA USA300 UAMS1516, followed by treatment with UA, nafcillin, a combination of both or vancomycin (positive control). (**a**) Lesion size (cm^2^) after 32 h and 56 h and (**b**) representative lesions at 56 h are shown. (**c**) CFU/g skin of MRSA recovered from skin lesions harvested from animals at 56 h time point. ELISA analysis of IL-1β (ng/g skin) levels recovered from skin lesions (**d**) or from serum (pg/mL) (**e**) harvested from animals after 56 h. Statistical analyses were performed by Tukey’s multiple comparison test (**** *p* < 0.001; *** *p* < 0.005; ** *p* <0.01; * *p* <0.05; ns, *p* >0.05).

Nafcillin was used instead of oxacillin because its activity has been well described before in this model [33] and it is considered as interchangeable with oxacillin based on the comparison of their pharmacological properties [34,35]. UA was selected as triterpenic acid because it was synergistic with oxacillin and ampicillin at lower concentrations than OA (see Figure 2). Moreover, it was also synergistic when combined with nafcillin against MRSA USA300 UAMS1182, while the combination of OA with nafcillin only led to additive effects (Table 2). Female C57bl6 mice were infected subcutaneously on their back with MRSA USA300 UAMS1182, for which the model has been established previously [36]. UA, nafcillin, a combination of both, vancomycin (positive control) or a vehicle (negative control) were administered subcutaneously to groups of mice immediately after MRSA infection and then every eight h for a total of 56 h (Figure 5).

The size (cm^2^) of the resulting necrotic skin lesions was measured after 32 and 56 h and is represented in panels a,b. Lesions were significantly smaller in the presence of UA after 32 h, although this effect was not maintained after 56 h. Administration of 100 mg/kg of nafcillin alone every 8 h significantly reduced the lesion size at both time points, and addition of 60 mg/kg of UA with nafcillin significantly improved the beneficial effect of nafcillin observed after 56 h. Residual lesions had statistically similar size in the group treated by the combination or by vancomycin. All treatments except UA significantly reduced the lesional MRSA burden (cfu/g skin; panel c). However, no statistically significant difference was observed between mice treated by nafcillin alone or combined with UA. Among the biological activities associated with UA, its anti-inflammatory effect is attributed to its ability to inhibit the immunoregulatory transcription factor NFκB in response to a wide variety of carcinogens and inflammatory agents [37,38]. We therefore measured levels of IL-1β, a key pro-inflammatory cytokine, in the skin and in the serum of infected animals, after 56 h of treatment (panels d,e). While nafcillin or UA alone did not affect IL-1β levels recovered from the skin, the nafcillin-UA combination and vancomycin to an even larger extend, caused a significant reduction. This effect was not observed in serum, where no treatment was effective in significantly altering the IL-1β levels.

We thus demonstrate here that synergy also takes place in vivo, using the combination of UA and nafcillin in a model of skin infection. Although UA did not increase the activity of nafcillin on bacterial burden, it improves its effect on the size of the lesion and the production of pro-inflammatory cytokine IL-1β in the skin. These benefits can nevertheless probably be ascribed to a synergy with the antibiotic, as UA alone was ineffective.

These two triterpenic acids have previously been shown to possess several biological activities including, among others, anti-inflammatory, anti-cancerous, or antiparasitic effects; they are able to modulate signaling pathways during disease development [39,40,41]. This lack of specificity and their reported toxicity in vitro could be a limitation of this study. However, the potential therapeutic interest of these compounds is highlighted by the fact that they are currently evaluated in a series of clinical trials for their metabolic, anti-inflammatory, or anti-cancer effects [40,42,43,44,45,46]. Of interest, published clinical trials (although still limited to Phase I), in which these compounds are administered orally or intravenously using liposomal formulations, did not evidence toxicity [42,43,44,45,46], further encouraging research on their therapeutic potentials in topic formulations to treat skin infections. Our results are also limited to the combination of UA and OA with β-lactams. Combinations with other classes of antibiotics have already been described as synergistic for glycopeptides, tetracyclines or macrolides [20,47] or additive for fluoroquinolones against different *Staphylococcus aureus* strains [48,49]. The mechanism of these interactions has not been elucidated yet but should implicate other mechanisms than those discovered here, though also possibly related to their interaction with membranes.

In spite of these limitations, our work brings important new pieces of information. We report for the first time the presence of high quantities of UA and OA in the leaves of *Vitellaria paradoxa* and demonstrate they are synergistic with β-lactams against MRSA. We partially elucidate the mechanism by which they revert resistance mediated by β-lactamases or PBP2A production. Moreover, we confirm the synergistic activity of UA with β-lactams in a murine model of subcutaneous MRSA infection without evidence of toxicity. Additional studies are now needed aiming at identifying their exact molecular target in the bacterial envelope as a way to orient the search of even more potent modulators of resistance from plants or obtain more effective derivatives by hemisynthesis.

## 3. Materials and Methods

### 3.1. Plant Material

Leaves and twigs of *Vitellaria paradoxa* C.F. Gaertn. (syn. *Butyrospermum parkii*) were collected in Benin (Parakou) in August 2012. A voucher specimen was identified and deposited at the Herbier National of Abomey-Calavi University in Benin, bearing the number AP2130.

### 3.2. Preparation of Extracts

Dried and powdered leaves or twigs (100 g) of *Vitellaria paradoxa* were extracted using a soxhlet apparatus with 700 mL of two solvents of increasing polarity (hexane and dichloromethane) for 8 h each. The extracts obtained were then evaporated to dryness under reduced pressure with a rotary evaporator at a temperature of 30 °C. We obtained 4.62 g and 1.83 g for the hexane and dichloromethane extracts respectively.

### 3.3. Reference Compounds

Ursolic (UA) (purity 91%) and oleanolic (OA) (purity 98%) acids were bought from AvaChem (San Antonio, TX, USA) and solubilized in DMSO. Ampicillin (potency 87.99%), oxacillin (potency 90%), nafcillin (potency 82%), gentamicin sulfate (potency 64.40%) and the β-lactamase inhibitor clavulanic acid (potency 50%) were obtained as microbiological standards from Sigma-Aldrich (St. Louis, MO, USA).

### 3.4. UA and OA Identification

The identification of compounds present in the crude extract was performed with a LC-MS/MS system consisting of a Thermo Accela pump, autosampler, photodiode array detector and Thermo Scientific LTQ orbitrap XL mass spectrometer (MASSMET platform) (Thermo Fisher Scientific, Waltham, MA, USA). The column used was a LiChroCART C_18_ 250 mm × 4.6 mm packed with 5 µm particles (Merck, Kenilworth, NJ, USA). The flow rate was 800 µL/min using a binary solvent system: Solvent A, HPLC grade water-acetonitrile (9:1) with 0.1% formic acid and solvent B, acetonitrile with 0.1% formic acid (0–5 min: 100% A, 70–85 min: 0% A, 86–96 min: 100% A). High-resolution MS was measured with APCI source in the negative mode. The following inlet conditions were applied: capillary temperature 250 °C, APCI vaporiser temperature 400 °C, sheath gas flow 20.00 u.a., auxiliary gas flow 5.00 u.a., sweep gas flow 5.00 u.a. Data acquisition and processing were performed with Xcalibur software (Thermo Fisher Scientific, Waltham, MA, USA).

### 3.5. UA and OA Quantification

The quantification of UA and OA from the crude extracts was performed according to a method adapted from the one described in PHARMEUROPA 2015 for the Common Selfheal fruit-spike [50]. We used an HPLC-UV system consisting of a Merck Hitachi pump, autosampler and a Waters UV detector (LambdaMax, model 481) (Merck, Kenilworth, NJ, USA). The column used was a Merck LiChroCART C_18_ 250 mm × 4.6 mm packed with 5 µm particles. The flow rate was 800 µL/min using an isocratic binary solvent system: Solvent A (20%), H_2_O pH 6 (NH_4_H_2_PO_4_ 0.04 M); solvent B (80%), Acetonitrile (ACN)/MeOH 40:35. Peaks were detected at 210 nm. A calibration curve was obtained for UA and OA methanolic solutions at concentrations ranging from 0.020 to 0.150 mg/mL (correlation coefficients (r^2^) of calibration curves >0.99). Plant extract solutions were prepared by dissolving 10 mg of extract in 10 mL of methanol. The injection volume used was 20 µL. To check the specificity of the method we used an LC-PDA-MS/MS system and the same LC conditions as those used for the quantification except that the pH buffer NH_4_H_2_PO_4_ 0.04 M had to be replaced by volatile CH_3_COONH_4_ 0.02 M. After having confirmed that the buffer change didn′t affect the elution profile with the UV detector, the specificity of the method was checked by comparing the mass and UV spectra at three different zones of the peaks with those of the respective standards.

### 3.6. Bacteria Strains

*Staphylococcus aureus* ATCC33591 (MRSA and β-lactamase producer; American Type Culture Collection, Manassas, VA, USA) was used as a reference strain. Four clinical isolates of MRSA were collected at the Universitair Ziekenhuis Brussel (UZ Brussel, Brussels, Belgium) in 2009. The strain VUB2 was isolated from a respiratory infection, VUB10 from a wound infection, and VUB20 and VUB30, from wound infections in diabetic patients. The homogeneous MRSA strain BCBPM073, derived from COL and carrying a single copy of PBP2 with an N-terminal superfast GFP fusion [26] was used for the PBP2 localization study. The strain MRSA USA300 UAMS1182 was used for the murine subcutaneous infection model [36].

### 3.7. Minimal Inhibitory Concentration (MIC) Determination

MICs were determined by broth micro-dilution method according to the guidelines of the Clinical and Laboratory Standards Institute in cation-adjusted Mueller-Hinton broth (caMHB) [51].

Briefly, bacteria were cultured on tryptic soy agar (TSA) (Difco, Richmond, CA, USA), incubated overnight at 37 °C and adjusted to a bacterial density of 10^6^ CFU/mL as starting inoculum. Triterpenic acids were solubilized in dimethylsulfoxide (DMSO) (Sigma, St. Louis, MO, USA) and then diluted in the nutrient broth to obtain a final concentration of 256 mg/L. Serial two-fold dilutions were made to obtain a concentration range from 0.25 to 128 mg/L and 7.81 to 500 mg/L respectively. After overnight incubation, 30 µL of a 0.02% resazurin (Sigma, St. Louis, MO, USA) solution was added to the wells (in order to facilitate the detection of bacterial growth) [10] and the plates were incubated during 1 h at 37 °C in the dark. The MIC corresponds to the lowest concentration of compound for which no metabolization of blue resazurin in pink resorufin was observed by sight. All tests were made in triplicate.

### 3.8. Fractional Inhibitory Concentration Indices (FICI) Determination

Fractional Inhibitory Concentration Indices (FICI) were determined by the checkerboard method in ca-MHB [52]. In a 96-well plate, the β-lactam antibiotic in the combination was serially diluted starting from a final concentration of 2× MIC along the ordinate. The triterpenic acid was serially diluted along the abscissa using 2× MIC as the highest final concentration. The bacterial suspension (final inoculum 0.5–1 × 10^6^ CFU/mL) was added to the wells.

After 20 h of incubation at 37 °C and addition of resazurin, MICs of antibiotics and triterpenic acid were determined as the lowest concentration that completely inhibited the growth of the organism.

Interactions between antibiotics were then evaluated using the FIC Indices, calculated as the sum of the Fractional Inhibitory Concentrations (FICs) as follows: FICI = FIC A + FIC C, where FIC A is MIC of the antibiotic in the combination/MIC of the antibiotic alone and FIC C is MIC of the compound in the combination/MIC of the compound alone [53]. The combination was considered as synergistic for FICI ≤ 0.5, additive for 0.5 < FICI ≤ 1, indifferent for 1 < FICI ≤ 4 and antagonistic for FICI > 4 according to the European Committee for Antimicrobial Susceptibility Testing [15]. All tests were made in triplicate.

### 3.9. Time-Kill Assay

Time-kill assays were performed as previously described [54] using ampicillin, oxacillin, UA, OA or the combination of the β-lactam antibiotic and a triterpenic acid, and the reference strain *S. aureus* ATCC33591. Briefly, an initial inoculum of 5 × 10^6^ CFU/mL in caMHB was used. Antibiotics were used as subMIC concentrations that did not impair bacterial growth at 24 h (64 and 16 mg/L for ampicillin and oxacillin respectively) and combined with four concentrations of triterpenic acids corresponding to their respective MIC, MIC/2, MIC/4 and MIC/8. The cultures were incubated at 37 °C and aliquots were removed at 0, 4, 8 and 24 h and serially diluted for CFU enumeration on TSA plates. Data were expressed as the change in log_10_ CFU from the initial inoculum after 24 h incubation.

### 3.10. Effect of Compounds on PBP2 Localization

Cultures of MRSA strain BCBPM073 carrying the GFP-PBP2 fusion were grown overnight in TSB and diluted 1:200 into 20 mL TSB. Cultures were incubated at 37 °C, with shaking (180 rpm), to exponential phase, corresponding to an OD_600 nm_ of approximately 0.4. Cultures were divided into 5 mL aliquots and incubated a further 30 min with 8 mg/L UA or 16 mg/L OA (concentration equivalent to their MIC). The same volume of solvent DMSO was added to the culture as a negative control, while 256 mg/L oxacillin (1× MIC) was used as a positive control. Cells were pelleted, suspended in Phosphate Buffer Saline (PBS) and observed by fluorescence microscopy on 0.1% PBS agarose pad. A Zeiss Axio Observer inverted microscope equipped with Photometric CoolSNAP HQ2 camera and Zen software was used for conventional microscopy. Structured illumination microscopy was performed using an Elyra PS.1 microscope (Zeiss, Oberkochen, Germany) equipped with a Plan-Apochromat 63 × 1.4 oil DIC M27 objective and a Pco.edge 5.5 camera. Images were acquired using five grid rotations, with grating 28 µM period for 488 nm laser (100 mW) and reconstructed using ZEN software (black edition, 2012, version 8.1.0.484) as described previously [55]. Fluorescence ratio (FR) were calculated as (a − c)/(b − c) where “a” corresponds to the fluorescence measured at the septum, “b” to the fluorescence of the peripheral membrane and “c” to the background fluorescence [56]; these measures were performed on conventional microscopy images using ImageJ software (public domain).

### 3.11. Effect of Compounds on PBP2 and PBP2A Expression

Cultures of MRSA strain BCBPM073 carrying the GFP-PBP2 fusion were grown overnight in TSB and diluted 1:200 into 200 mL TSB. Cultures were then prepared following the same conditions as those used for the PBP2 localization experiment. Protein extraction and western blotting were performed as previously described, with minor modifications [57]. Cells were pelleted, suspended in PBS, and lysed using glass beads in a Fast Prep FP120 (Thermo Electro Corporation, Waltham, MA, USA). Cells were separated from the glass beads by centrifugation for 1 min at 4200 rpm, and unbroken cells were removed by centrifugation for 15 min at 13,000 rpm. Total protein content was quantified using the BCA protein assay kit (Pierce, Waltham, MA, USA) and 50 mg of protein extract were loaded in a 10% SDS-PAGE gel. After separation at 80 V for 4 h, samples were transferred to a Hybond-P Polyvinylidene difluoride (PVDF) membrane (GE Healthcare, Little Chalfont, UK) using a semidry transfer cell (BioRad, Hercules, CA, USA), cut between the 70 kDa and 100 kDa markers (to separate GFP-PBP2 and PBP2A containing regions) and blocked for 1 h with 5% milk in PBST (0.5% Tween 20 in phosphate buffered saline). Membranes were incubated overnight with 1:5000 of polyclonal anti-PBP2 or 1:500 of anti-PBP2A (Slidex MRSA detection, BioMerieux, Marcy-l’Étoile, France) antibodies, washed with PBST the following day, and incubated for 1 h with 1:50,000 of HRP-conjugated goat anti-mouse or 1:50,000 of HRP-conjugated goat anti-rabbit antibodies, respectively. Bands were visualized using the ECL Plus Western blotting detection kit (GE Healthcare, Little Chalfont, UK) and Chemidoc XRS + Imaging System (BioRad, Hercules, CA, USA).

### 3.12. Evaluation of β-Lactamase Activity using the Nitrocefin Test

Nitrocefin is a chromogenic cephalosporin that can be used as a substrate for β-lactamase activity [58]. This yellow cephalosporin generates a red metabolite upon hydrolysis of its β-lactam ring by β-lactamases, the absorbance of which can be followed overtime. Bacteria were grown overnight in caMHB. A fresh suspension was prepared (OD_620 nm_ = 0.1) and incubated for 4 h at 37 °C and 130 rpm until exponential phase of growth (OD_620 nm_ = 0.5). Aliquots of bacteria (2 mL) were then incubated for 10 min in the presence or absence of UA or OA at appropriate concentrations after which 180 µL of each sample was mixed with 20 µL of a 0.05% nitrocefin solution (CalBiochem/EMD Millipore, Billerica, MA, USA). The changes of absorbance were recorded at 486 nm during 30 min at 37 °C SpectraMax microtiter plate reader (Molecular Devices LLC, Sunnyvale, CA, USA). Gentamicin sulfate and clavulanic acid were used as negative and positive controls respectively.

In order to distinguish between a direct inhibition of enzymatic activity and an indirect effect on β-lactamase activity dependent on their anchoring in the bacterial membrane of living bacteria, the same experiment was also performed on bacterial lysates. Bacteria in exponential growth (DO_620 nm_ = 0.5) were centrifuged 7 min at 4000 rpm. The supernatant was removed and the bacterial pellet was suspended in 500 µL of a detergent buffer, B-PER Bacterial Protein Extraction Reagent (Thermo Electro Corporation, Waltham, MA, USA). This lysate was resuspended in the initial volume of medium, added by the tested compounds and then by nitrocefin as described above. All tests were made in triplicate.

### 3.13. Murine Model of Subcutaneous Infection

MRSA USA300 UAMS1182 was grown to mid-log phase in THB (A_600_ 0.4), washed twice in PBS, and resuspended in PBS. Shaved 7-week-old female C57bl6 mice were anesthetized using isoflurane and injected in the mid-back with 50 µL containing approximately 1 × 10^7^ CFU. A first experiment was made with 5 mice per group and a second one with 8 mice per group. Group one received only the vehicle (Tween/ethanol/PBS 5:5:90), group two received 60 mg/kg of UA, group three received 100 mg/kg for nafcillin and group four received the combination of UA (60 mg/kg) and nafcillin (100 mg/kg). Those groups were treated immediately after the inoculation of bacteria and then every 8 h for three days. All treatments were administered subcutaneously close to the infection site. A fifth group receiving vancomycin (25 mg/kg) twice a day was used as a positive control. Abscesses were photographed after 36 and 52 h, and the area was quantified using Image J. Abscess area was defined as the zone of leukocytic infiltration including the contained zone of dermonecrosis. On day 3, abscesses were excised and homogenized in PBS prior to serial dilution and drop plating. Abscess burden was calculated as number of CFU per g of skin. The inflammatory cytokine IL-1β level was quantified in the skin and in the serum by ELISA (R&D Systems, Minneapolis, MN, USA) according to the manufacturer’s instructions.

Animals were maintained in accordance with the American Association for Accreditation of Laboratory Animal Care Criteria, and the above-described studies were approved by the Animal Care and Use Committee (IACUC) (Protocol S00227M) of the University of California, San Diego, CA, USA.

### 3.14. Statistical Analysis

All data were analyzed using GraphPad Prism 6 Software (GraphPad Software, San Diego, CA, USA).

## Figures and Tables

**Table 1:** Antimicrobial activity of ursolic acid (UA), oleanolic acid (OA) and β-lactams on MRSA strains and FIC index (FICI) of their combinations.

Strain	MIC (mg/L)				FICI			
	UA	OA	Ampicillin	Oxacillin	Ampicillin-UA	Ampicillin-OA	Oxacillin-UA	Oxacillin-OA
**ATCC 33591**	16	32	64	32	0.38–1	0.31–1	0.25–1	0.16–1
**VUB 2**	8	64	32	64	0.50–1	0.25–1	0.31–1	0.18–1
**VUB 10**	8	64	128	128	0.37–1	0.31–1	0.31–1	0.18–1
**VUB 20**	8	64	64	256	0.37–1	0.25–1	0.50–1	0.37–1
**VUB 30**	16	128	32	512	0.56–1	0.37–1	0.50–1	0.25–1

**Table 2:** Antimicrobial activity of ursolic acid (UA), oleanolic acid (OA), oxacillin (OXA) and nafcillin (NAF) and FIC index of their combinations on the MRSA USA300 UAMS1182 strain.

Compound	MIC (mg/L)	FICI	
		OXA	NAF
**UA**	64	0.31–1	0.28–1
**OA**	>128	0.50–1	0.56–1
**OXA**	32	-	-
**NAF**	32	-	-

## Supplementary Materials

The following are available online, Figure S1: Isobolograms showing the in vitro interaction between the triterpenic acids (UA or OA) and ampicillin or oxacillin on the reference strain ATCC33591 and on the four clinical MRSA strains (VUB 2,10,20 & 30), Figure S2: Time-kill curves against MRSA ATCC33591 for the combination of ampicillin or oxacillin with ursolic acid, Figure S3: Time-kill curves against MRSA ATCC33591 for the combination of ampicillin or oxacillin oleanolic acid.
