# Supplementary material for: Synergy between Ursolic and Oleanolic Acids from Vitellaria paradoxa Leaf Extract and β-Lactams against Methicillin-Resistant Staphylococcus aureus: In Vitro and In Vivo Activity and Underlying Mechanisms
Source: Molecules. 2017 Dec 16;22(12):2245. doi: 10.3390/molecules22122245 (PMC6149719; doi:10.3390/molecules22122245)

Supplemental material

Figure 1S. *Isobolograms showing the in vitro interaction between the triterpenic acids (UA ■ or OA ●) and ampicillin (A) or oxacillin (B) on the reference strain ATCC33591 and on the four clinical MRSA strains (VUB2,10,20 & 30). Each point corresponds to the fractional inhibitory concentration (FIC) of the triterpenic acid (Y axis) and the FIC of the antibiotic (X axis). Represented data are the mean ± SD of three individual experiments.*

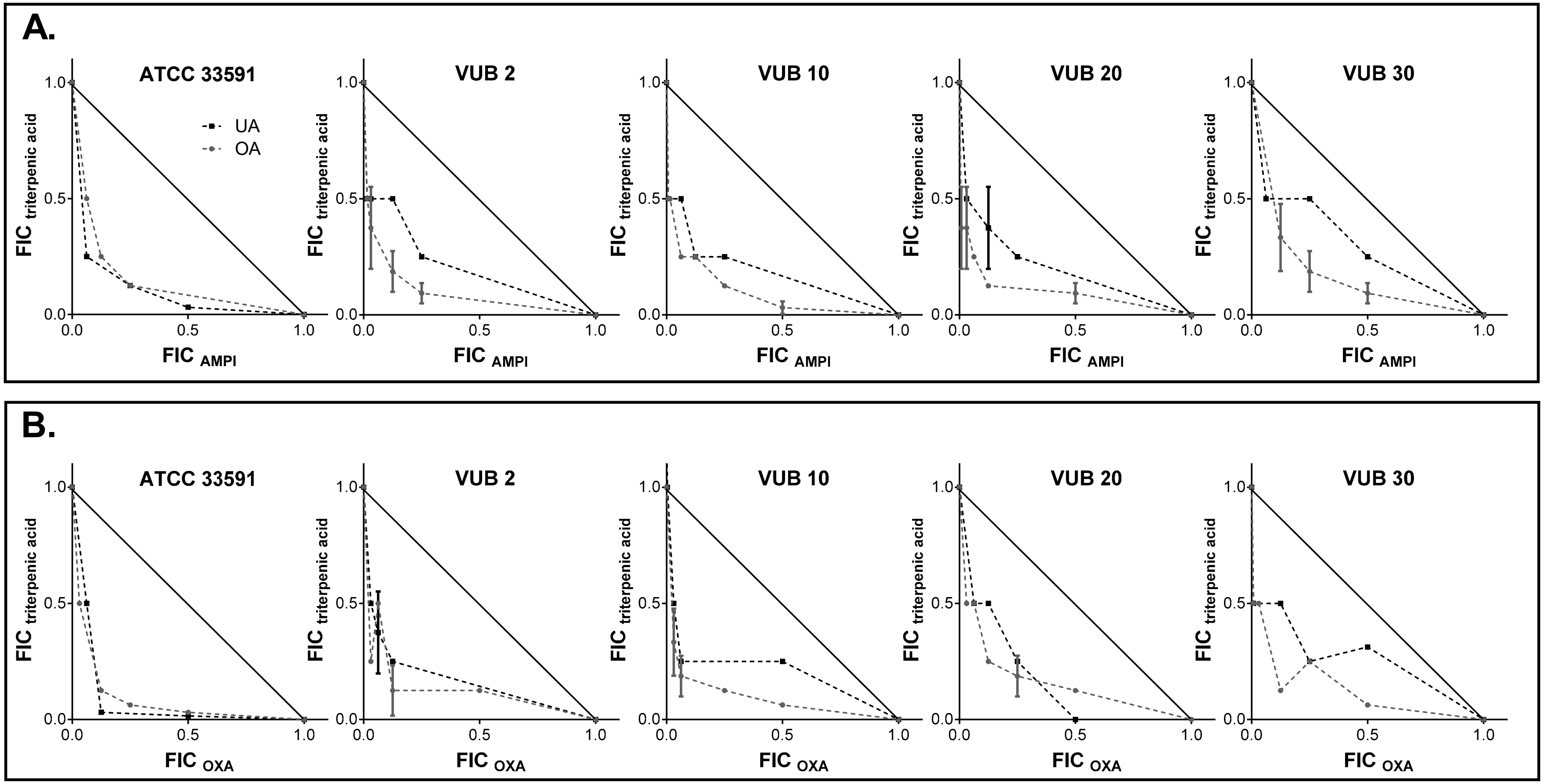

Figure 2S. *Time-kill curves against MRSA ATCC33591 for the combination of ampicillin (A.) or oxacillin (B.) with four different concentrations of ursolic acid corresponding to the MIC, ½ x MIC, ¼ x MIC or 1/8 x MIC. Represented data are the mean ± SD of 3 experiments performed in duplicate.*

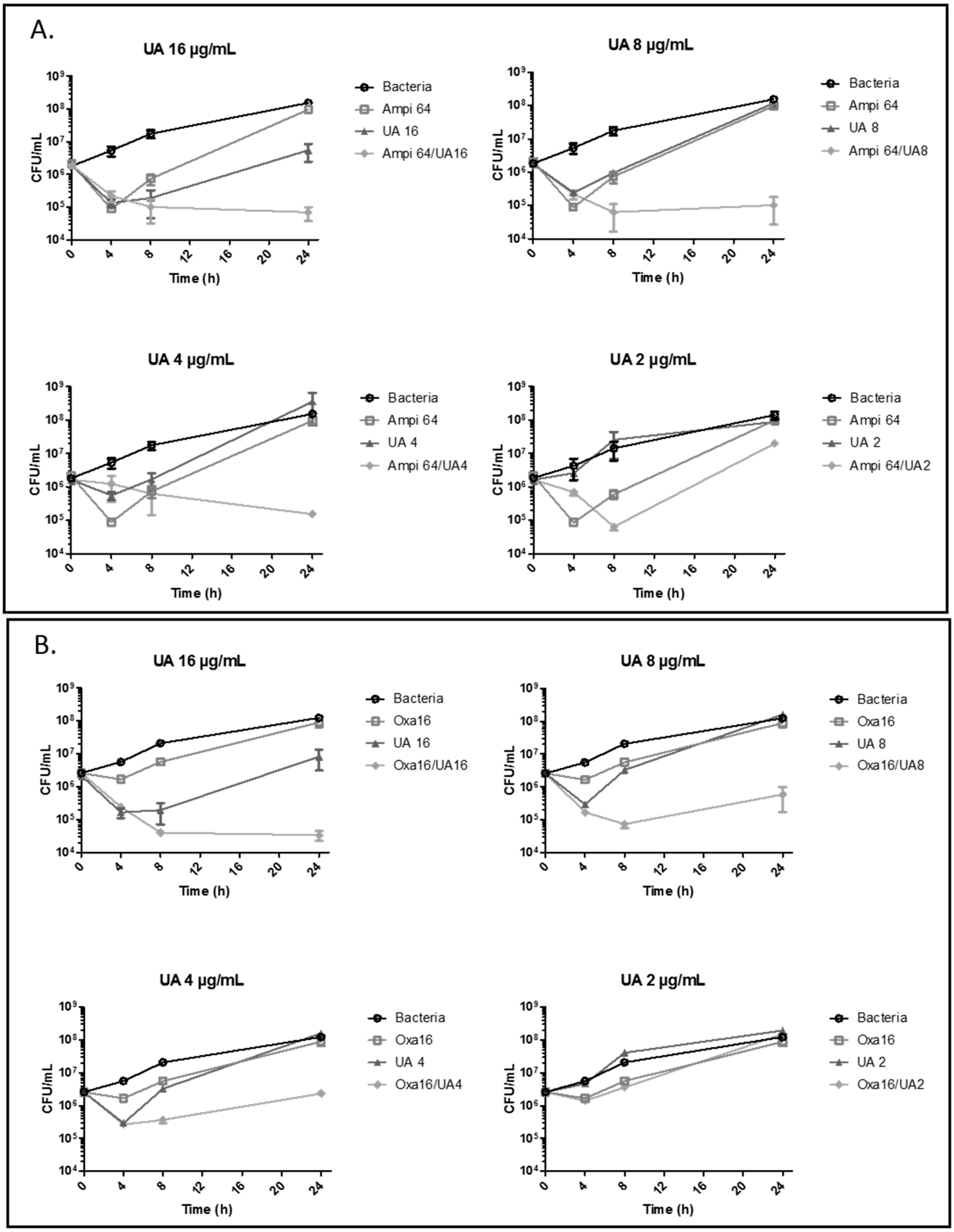

Figure 3S. *Time-kill curves against MRSA ATCC33591 for the combination of ampicillin (A.) or oxacillin (B.) with four different concentrations of oleanolic acid corresponding to the MIC, ½ x MIC, ¼ x MIC or 1/8 x MIC. Represented data are the mean ± SD of 3 experiments performed in duplicate.*

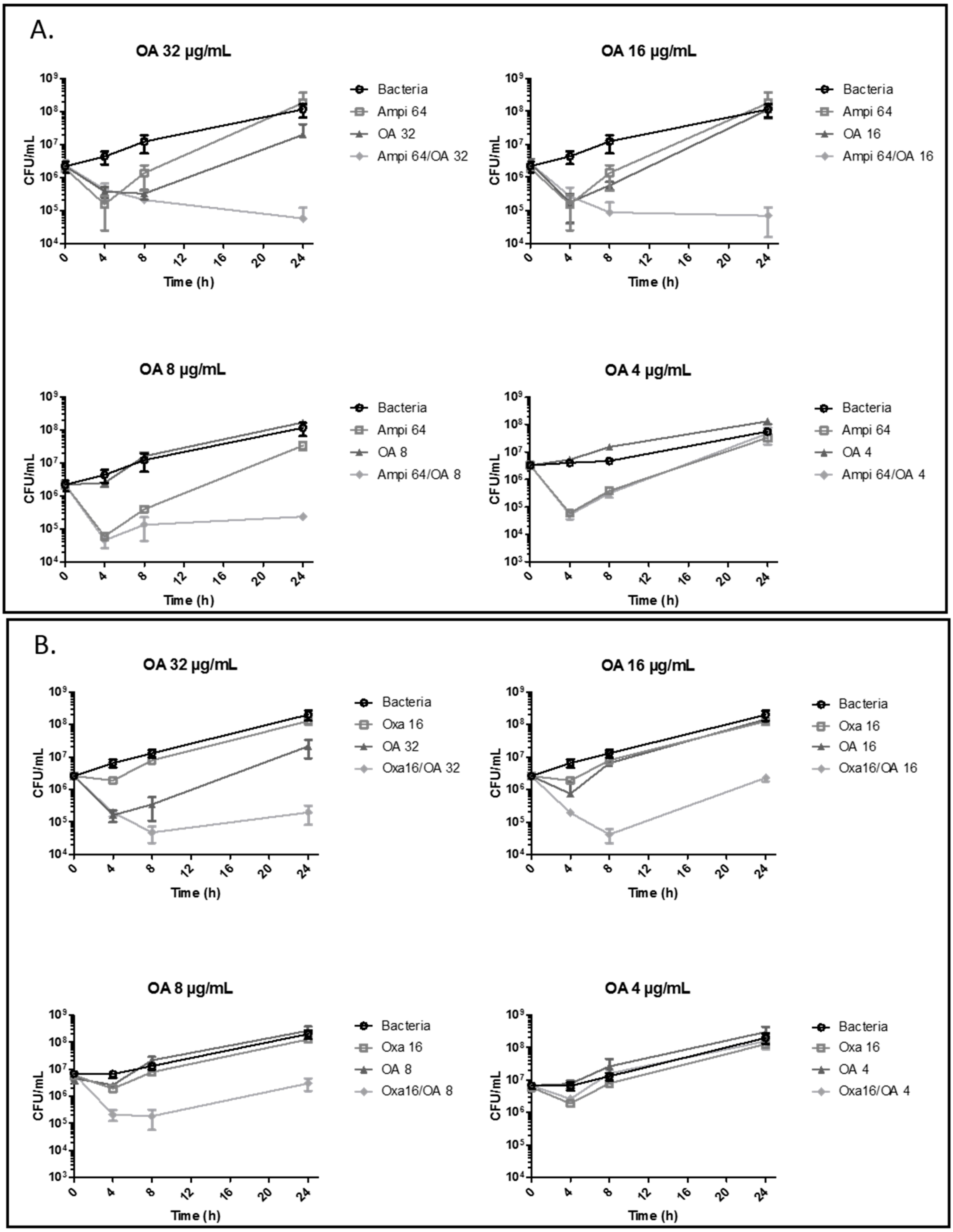

Supplement: Supplementary file 1 [file molecules-22-02245-s001.pdf]
